# Effect of Waste Glass Incorporation Methods on the Physical, Mechanical and Microstructural Properties of Cementitious Binders

**DOI:** 10.3390/ma19071346

**Published:** 2026-03-28

**Authors:** Jurgita Malaiškienė, Karolina Bekerė, Jelena Škamat

**Affiliations:** Laboratory of Composite Materials, Faculty of Civil Engineering, Vilnius Gediminas Technical University, Sauletekio Av. 11, 10223 Vilnius, Lithuania; karolina.bekere@vilniustech.lt (K.B.); jelena.skamat@vilniustech.lt (J.Š.)

**Keywords:** glass waste from household and electronic appliances, different mixing types, cement hydration, structure, physical and mechanical properties

## Abstract

In previous studies, it was established that replacing cement with dispersed glass from various electronic and household devices is challenging due to the formation of agglomerates in the mixture. Therefore, this study addresses this problem by applying different methods for incorporating dispersed glass: mixing in a conventional Hobart-type mixer, mixing dry components in an intensive Eirich-type mixer, and dispersing the glass particles in water using ultrasonic treatment. Using these 3 glass waste incorporation methods, the properties of hardened cement paste—density, compressive strength, phase composition, and microstructure—were compared. The effects of 4 types of glass (from television screens, washing machines, fluorescent lamps, and solar panels) were analysed. The results showed that lamp glass dispersed in water with ultrasound showed the best performance, while for the other glass types, intensive mixing was more effective. Under these conditions, the compressive strength of the samples increased by up to approximately 24%, and a denser microstructure was obtained compared to other glass incorporation methods.

## 1. Introduction

As industrial production and technological processes accelerate, humanity generates an increasing quantity of materials that are difficult to decompose and turn into waste at the end of their life cycle [1]. The disposal of large amounts of waste in landfills removes materials from economic circulation, pollutes the environment, and contributes to global warming [2,3]. One of the waste materials produced is glass [4,5,6], which does not degrade due to environmental factors, is resistant to corrosion, bacteria, acids, and salts, and can take more than several hundred years to decompose [7,8,9]. The biggest problem is that there are many types of glass with different chemical compositions, as well as different physical and mechanical properties [10,11]. This complicates the recycling of non-container glass, as different types have different melting temperatures depending on their colour [12,13] and may contain harmful heavy metals, such as lead [14]. In addition, glass is often contaminated or difficult to separate from other materials. For example, solar panel glass is bonded with an EVA (ethylene-vinyl acetate) film [15].

Another problem in the construction industry is that cement production emits the highest amount of CO_2_ of all industries due to the high temperatures (up to 1450 °C) and large amounts of CaCO_3_ used [16,17]. To solve this problem, it is recommended that cement materials be replaced with waste materials wherever possible [18,19]. Milled glass waste containing more than 60% SiO_2_ [20] can act as pozzolanic material that participate in the secondary hydration process in which calcium silicate hydrates are formed [21,22]. Scientists have found that improved fineness of glass particles generally increases pozzolanic reactivity [23,24].

Scientists claim that fine, undissolved glass particles in cement mixtures can agglomerate in the binder. These structures can increase porosity and cause microcracks due to poor adhesion between the smooth glass surface and the cement matrix [25,26]. However, fine glass particles in cementitious mixtures can increase compressive strength. However, they agglomerate and can alter the properties of the binder if the mixture is not properly prepared [27,28,29]. Agglomeration can be reduced with additives, such as additional fibres or superplasticizers [30,31,32]. Generally, there is no consensus on the optimal size of glass particles in cementitious mixtures because it depends on the type of glass used and the way the mixture is prepared [33,34]. W. Dong [8] reviewed scientific articles on glass waste in cementitious materials and found that the results vary; one reason is the suboptimal preparation or dispersion of the mixture. S. Poudel et al. [35] concluded that finer glass in cement mixtures can prolong the setting time when waste glass is added to concrete as a partial cement substitute. However, in some cases, the reduction in setting time may be due to the fact that glass particles are finer than cement particles. André L. S. Patriota et al. [36] and our previous work [37] found that agglomerates formed in hardened cement paste samples with fine glass waste when the particle size was less than 45 µm, which demonstrated the importance of the mixing methodology.

To reduce porosity and the quantity of agglomerates in cement mixtures by replacing cement with glass waste, different methods of preparing glass and cement mixtures can be used, such as different mixers, dispersion, and different mixing times. There are not many sources in the literature that investigate the dispersion mixing method specifically for cement mixtures with glass. E. Šerelis et al. conducted a detailed study on the high-frequency ultrasonic dispersion mixing of ultra-high-performance concrete (UHPC) cement paste with bottle glass which average particle size is 25.8 µm [38]. They found that this method effectively deagglomerates Portland cement and glass and silica fume particles, accelerating the hydration of clinker minerals and intensifying the pozzolanic reaction. The study showed that this method increased the degree of Portland cement hydration from 26% to 35%. Additionally, combining high-frequency ultrasonic dispersion with thermal treatment increased the compressive strength from 141.1 MPa to 177.8 MPa. E. Šerelis [39] also investigated the properties of alumina-based concrete using high-frequency ultrasonic dispersion and liquid glass. They found that replacing Portland cement with glass powder by up to 80% significantly slows the hydration process, resulting in a 77% decrease in compressive strength. However, applying 60 s of high-frequency dispersion treatment to a mixture in which 30% of the cement was replaced with glass waste resulted in a return to the initial compressive strength of 3.1 MPa. Combining dispersion with liquid glass produced additional improvements: compressive strength increased by 87%, density increased by 32%, and porosity decreased significantly.

This is a more popular mixing method for cementitious materials in mixers. For example, T. Azis [40] observed that when incorporating a substantial quantity of glass powder (>65%) into an alkaline hybrid binder activated with sodium carbonate and sodium citrate, the binding materials were dry-mixed for approximately one minute in a Hobart mixer during the preparation of paste samples. Microstructural analysis revealed a dense cement–glass matrix with a phase of riverside, resulting in significantly improved mechanical properties. V. Vaitkevičius et al. [41] investigated the effect of glass powder on the properties of UHPC. They prepared mixtures using an intensive mixing Eirich R02 mixer, mixing all materials dry first. The highest compressive strength (221 MPa) was found in a mixture where 100% of the quartz powder was replaced with glass powder and silica fume was used simultaneously. When silica fume was completely replaced with glass powder, however, the strength decreased to 153 MPa. This result confirms the limited pozzolanic activity of glass powder compared to silica fume. Microstructural analysis revealed that, in mixtures containing glass powder, macropores disappeared due to an alkaline silicate reaction and the dominant porosity shifted to the micro- and nanoscale. E. Šerelis et al. [42] prepared UHPC mixtures with bottle waste glass in which 100% of the quartz powder was replaced with glass powder. The dry materials were first mixed in a laboratory mixer Eirich R02 (Maschinenfabrik Gustav Eirich GmbH & Co KG, Hardheim, Germany) and then in an industrial mixer HPGM 1125 (Wiggert & Co. GmbH, Karlsruhe, Germany). Different amounts of steel fibres were mixed into the new UHPC mixture, increasing the flexural strength about fivefold, from 6.7 MPa to 36.2 MPa. The experiment also showed that such glass powder can replace silicon dioxide fume without reducing the mechanical properties.

The scientists [43] continued their research on the UHPC mixture, which was created using two different mixing systems. The first system was an intensive Eirich R02 mixer, and the second system involved processing the mixture in a Zyklos ZZ50HE rotary mixer (ZYKLOS MISCHTECHNIK GmbH, Recklinghausen, Germany), which is similar to those used in conventional concrete plants. The study showed that preparing UHPC with a W/C ratio of 0.3 is possible, even when integrating a large quantity of micro steel fibres (up to 147 kg/m^3^) into the mixture. The resulting concrete demonstrated significant improvements in mechanical properties: compressive strength increased from 116 MPa to 150 MPa and flexural strength increased significantly. K.H. Hoang et al. [44] also used the intensive mixing with Eirich-type mixer and found that, as the aggregate fraction decreases, the number of particles and their total surface area increase. At the same time, the plastic viscosity, self-flowability, and strength of the concrete improve significantly. These changes are associated with denser packing of the particles, smaller voids, and thicker paste layers around the particles. T. Hemalatha et al. [45] found that the mixing procedure, type of mixer, and other parameters, such as mix proportions, water-to-powder ratio, and mineral and chemical additives, significantly influence the properties of fresh and hardened concrete.

The available studies demonstrate that the application of additional dispersion techniques can significantly improve the homogeneity of cement-based mixtures and improve the hydration and mechanical performance of the resulting materials. However, the use of such approaches for cementitious systems containing glass waste has been investigated only to a limited extent (mainly in soda-lime glass), while the reported results, obtained using different techniques and experimental conditions, remain fragmented, complicating direct comparison and preventing a systematic understanding of how dispersion methods influence the behaviour of cement-based systems containing various glass waste.

The present work extends the investigation to four different types of glass waste originating from television screens, washing machines, fluorescent lamps, and photo-voltaic solar panels, which differ significantly in SiO_2_ content, the type and amount of secondary components, solubility, and other properties.

Three different approaches—conventional mixing, intensive mixing, and pre-dispersing of glass in water—were studied under identical laboratory conditions, providing not only quantitative material characteristics representing the effectiveness of different dispersion approaches but also contributing to a better understanding of how the nature of waste glass affects its interaction with cement matrix and the resulting hydration and hardening processes.

## 2. Materials and Methods

### 2.1. Materials

In this study, Portland cement CEM I 42.5R (AB Akmenės cementas, Naujoji Akmenė, Lithuania) was used with different types of glass waste (UAB Atliekų tvarkymo centras, Vilnius, Lithuania), including glass from discarded television screens (TV), washing machines (WM), fluorescent lamps (FL), and photovoltaic solar panels (SP). These glass types were chosen because of their very different chemical compositions (Table 1) and because they are in large amounts in waste separation landfills. Initially, each type of glass cullet was milled separately in a laboratory-scale ball mill using steel balls as the grinding bodies. The milling process was conducted for 2 h at a batch load of 10 kg, maintaining a constant ball-to-powder ratio. The corresponding particle densities were 2.95 g/cm^3^ (TV), 2.27 g/cm^3^ (WM), 2.71 g/cm^3^ (FL), and 2.50 g/cm^3^ (SP). The electrical conductivity values of the different glass types were also recorded. The values were 1.55 mS (TV), 6.23 mS (WM), 8.21 mS (FL), and 2.12 mS (SP). For all mixtures, a constant water-binder ratio (W/B) of 0.35 was used, and 30% of the cement mass was replaced with the selected waste glass materials. In control mix was only cement and water.

A detailed examination of the chemical composition of different types of glass reveals significant variations. The most pronounced differences are observed in the SiO_2_ content: TV glass contains only about 50%, whereas FL and SP glass contain about 70%, and WM glass exceeds 81%. There are also substantial differences in the levels of Na_2_O, K_2_O and CaO. The highest concentrations of heavy metals were identified in TV glass. Due to these compositional differences, it is expected that different types of glass will have distinct effects on cement hydration processes and other material properties.

**Figure 1:**
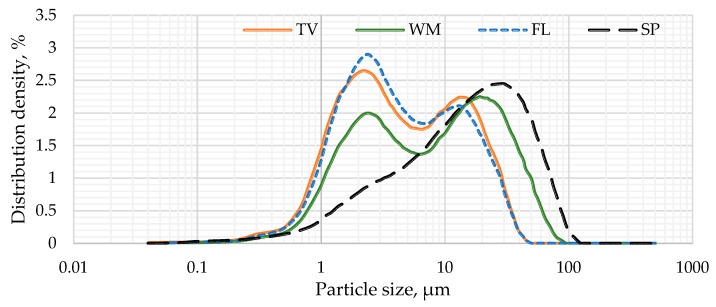
Different glass waste type particle size distribution.

As illustrated in Figure 1, the particle size distribution of the various types of glass after the same time of milling indicates that the mean particle size for glass waste was determined to be 7.2 µm (TV), 12.9 µm (WM), 7.0 µm (FL), and 20.8 µm (SP). An analysis of the particle size distribution curves reveals that the particle sizes of the TV, WM, and FL types of glass, when ground for an equivalent duration, exhibit negligible differences, indicating that the mechanical resistance of this glass is comparable. Greater disparities were identified in the SP glass, where the average particle size is approximately 21 µm.

**Figure 2:**
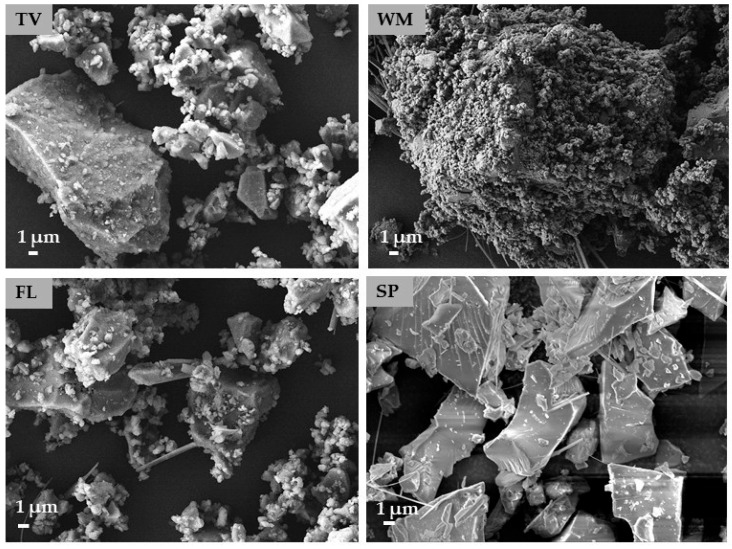
SEM images of different glass waste types.

The SEM images (Figure 2) reveal clear differences in particle morphology among glass types. The samples obtained from TV and FL glass consist of irregular, angular particles with rough surfaces and noticeable agglomeration of fine grains. WM is characterised by dense agglomerates of very fine particles with a predominance of needle-like crystalline structures that form a compact and highly clustered morphology. In contrast, SP exhibits larger, plate-like particles with smoother surfaces and well-defined edges. These observations suggest that the glass type exerts a significant influence on the morphology of particles and the characteristics of their surfaces after the same milling process.

### 2.2. Test Methods and Preparation of Samples

The chemical composition of glass waste (GW) was determined by XRF analysis using a Rigaku Primus IV instrument (Rigaku, Tokyo, Japan). The granulometric composition of GW was analysed using the ultrasonic dispersion method in an aqueous solution with the CILAS 1090 instrument (CILAS, Odelzhausen, Germany). The density of glass particles was established using a Helium (gas) pycnometer AccuPyc II 1340 (Micromeritics Instrument Corporation, Norcross, GA, USA). Electrical conductivity is determined using Metterler-Toledo MPC 227 (Mettler-Toledo GmbH, Leicester, UK) device with InLab 730 electrode.

**Figure 3:**
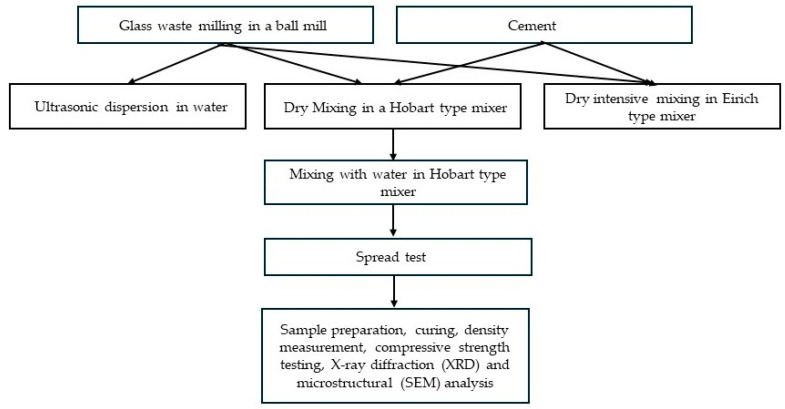
The experimental scheme.

The experimental scheme is presented in Figure 3.

In the experiment (Figure 3), 30% of the cement was substituted with the previously mentioned waste glass. The dry raw materials were mixed using three different methods: (1) in a Hobart mixer (2 min dry, 3 min with H_2_O), (2) intensive Eirich mixer (5 min dry) and Hobart mixer (3 min dry, 3 min with H_2_O), (3) dispersed 150 g glass in 175 g H_2_O with ultrasound and Hobart mixer (3 min).

Ultrasonic dispersion was performed in water with the required amount of glass for the mixture. The process was carried out for 1 min at a frequency of 22 kHz. The spread of the mixture was evaluated according to the method described in LST EN 12706. The samples were prepared using metal moulds with dimensions of 40 × 40 × 40 mm. Subsequent to a 24 h curing period at a temperature of 20 ± 1 °C and a relative humidity of 95% within moulds, the samples were demoulded and then subjected to water-based curing for a duration of 7, 28, and 90 days.

After this curing period, the density and compressive strength of the samples were evaluated. The evaluation involved analysis of 3 samples of each composition at each designated time point. The density was calculated from the dimensions measured with a 0.01 mm accuracy, and the mass was measured with a 0.01 g accuracy. The compressive strength of the samples was determined using the Tinius Olsen H200 KU hydraulic press (Tinius Olsen, Orlando, FL, USA).

For the purpose of phase analysis, the DRON-7 diffractometer (Bourevestnik, Inc., Saint Petersburg, Russia) was utilised, with an X-ray wavelength of λ = 0.1541837 nm (Cu-Kα). The X-ray diffraction curves were recorded at angular intervals ranging from 4° to 60°.

The microstructure of the binder samples at 7 and 28 days was analysed by scanning electron microscopy Thermo Fisher Scientific Quattro S (Thermo Fisher Scientific, Brno, Czech Republic). Prior to analysis, the samples underwent a gold coating process through the method of vacuum evaporation.

## 3. Results and Discussion

### 3.1. Influence of Different Mixtures Preparation Methods and Waste Glass Types on Binder Paste Workability

**Figure 4:**
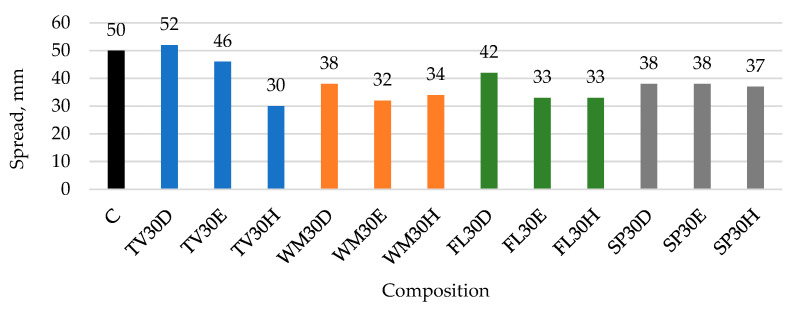
The impact of different mixing methods and GW types on the spread of cementitious mixtures.

The effect of the mixing method on the spread of the mixture was found to be significant, especially for the TV glass composition (Figure 4). The samples prepared by dispersing the glass in water with ultrasound exhibited the highest spread values (52 mm), which were comparable to the spread values of 50 mm observed for the control mix. These results may be attributed to a more homogeneous distribution of glass particles within the mixture and reduced agglomeration of TV glass particles in the cement matrix, leading to fewer air pores and improved workability. Previous research [34] has also shown that the dispersion and distribution of fine particles in cementitious systems significantly influence fresh-state properties such as flowability and spread, with a better dispersion typically resulting in improved rheological behaviour. The authors [46] demonstrate that improved dispersion and optimised particle packing enhance the rheological properties of cementitious materials, as particle distribution directly influences inter-particle spacing and flow behaviour in fresh suspensions. Intensive mixing of the TV composition in an Eirich mixer resulted in a slightly lower spread (46 mm); however, it was still approximately 1.5 times higher compared to conventional mixing in a Hobart mixer (30 mm). No pronounced increase in water demand associated with the larger specific surface area of glass was observed after additional dispersing, which would otherwise have reduced the spread of the paste. This may be attributed to the low solubility of TV glass (electrical conductivity 1.55 mS).

The WM and FL samples, which contained glass powders with a particle size comparable to that of TV, demonstrated a different response to additional dispersion. Intensive mixing did not improve the paste spread and, in the case of WM, even reduced it from 34 mm (WM30H) to 32 mm (WM30E), while ultrasonic dispersion in water resulted in a moderate increase in spread. The presence of irregular, angular particles and agglomerates (FL, and especially WM) could increase the friction between particles and the water demand, thus reducing the spread. In particular, the dense clusters and needle-like structures present in WM hinder particle mobility and limit paste spread. In contrast, smoother and more regular plate-like particles (SP) could reduce internal friction and facilitate improved particle rearrangement, thereby leading to an improvement in spread after dry glass incorporation methods. This behaviour can also be attributed to the significantly higher solubility of these glasses (6.23 mS for WM and 8.21 mS for FL) and to specific interfacial processes occurring in the glass–cement interaction zone. The dissolution of glass in aqueous solutions is a complex process involving the breakdown of Si–O–Si bonds within the glass structure, hydration, ion exchange, and re-condensation of dissolved silicate species, leading to the formation of a hydrated amorphous gel layer on the glass surface [26].

Intensive mixing disrupts the agglomerates, resulting in improved particle distribution and an increased effective surface area. However, gel layers formed upon contact with water can act as a “glue”, reducing workability and counteracting the positive effect of improved dispersion. It has been shown [47] that gel-like structures formed in the early stages of hydration strongly affect the rheology of cement pastes: they promote interparticle bridging, increase yield stress and plastic viscosity, improve structural build-up (thixotropy), and ultimately reduce workability during the initial dormant period. When the powders are first dispersed in water (WM30D and FL30D), dissolution occurs prior to contact with cement particles. Under ultrasonic treatment, the initially formed gel layers may be partially disrupted and more uniformly distributed in the aqueous phase. Subsequent mixing with cement therefore results in a more homogeneous system and improved paste spread.

It has been reported [48] that the thickness of the gel layer formed on dissolving glasses can vary from nanometres to microns, depending not only on leaching time and conditions but also strongly on glass composition. This factor, together with differences in particle morphology, may explain the slight variation in spread values observed between WM and FL compositions.

For SP glass, the most similar spread results across mixing methods can be linked to its comparatively coarser particle size (20.8 µm) and relatively homogeneous surface morphology and one of the lowest solubilities (electrical conductivity 2.12 mS).

### 3.2. Influence of Different Mixtures Preparation Methods and Waste Glass Types on the Binder Density and Compressive Strength

**Figure 5:**
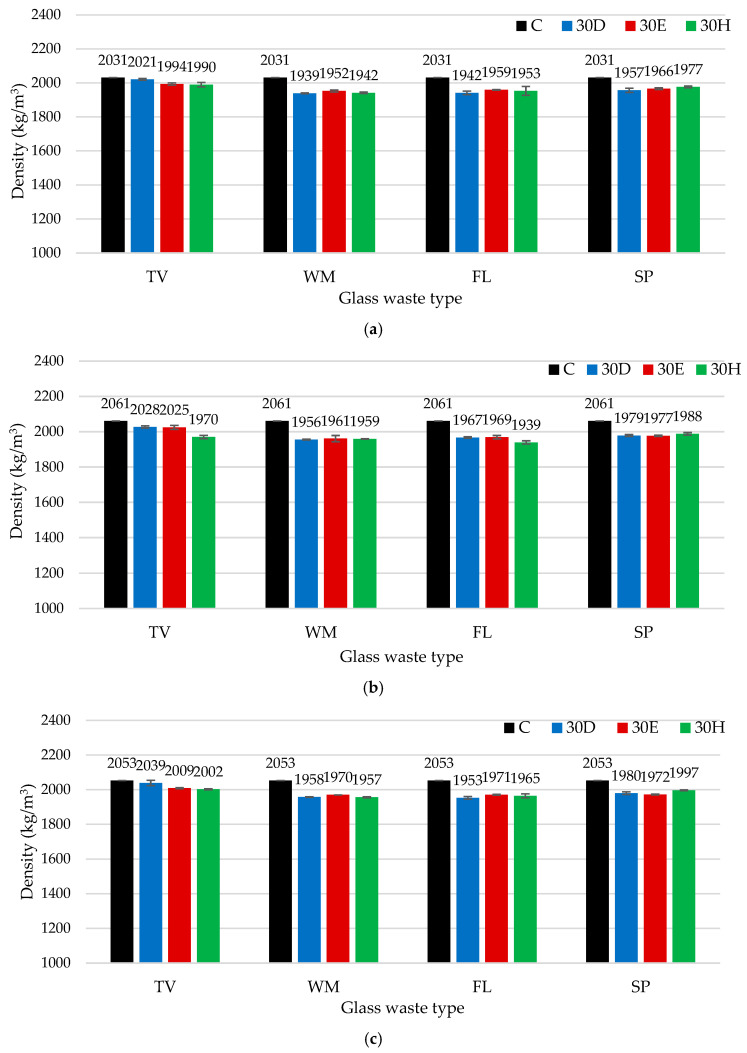
The impact of different mixing methods and GW types on density: (**a**) after 7 days, (**b**) after 28 days, and (**c**) after 90 days.

**Figure 6:**
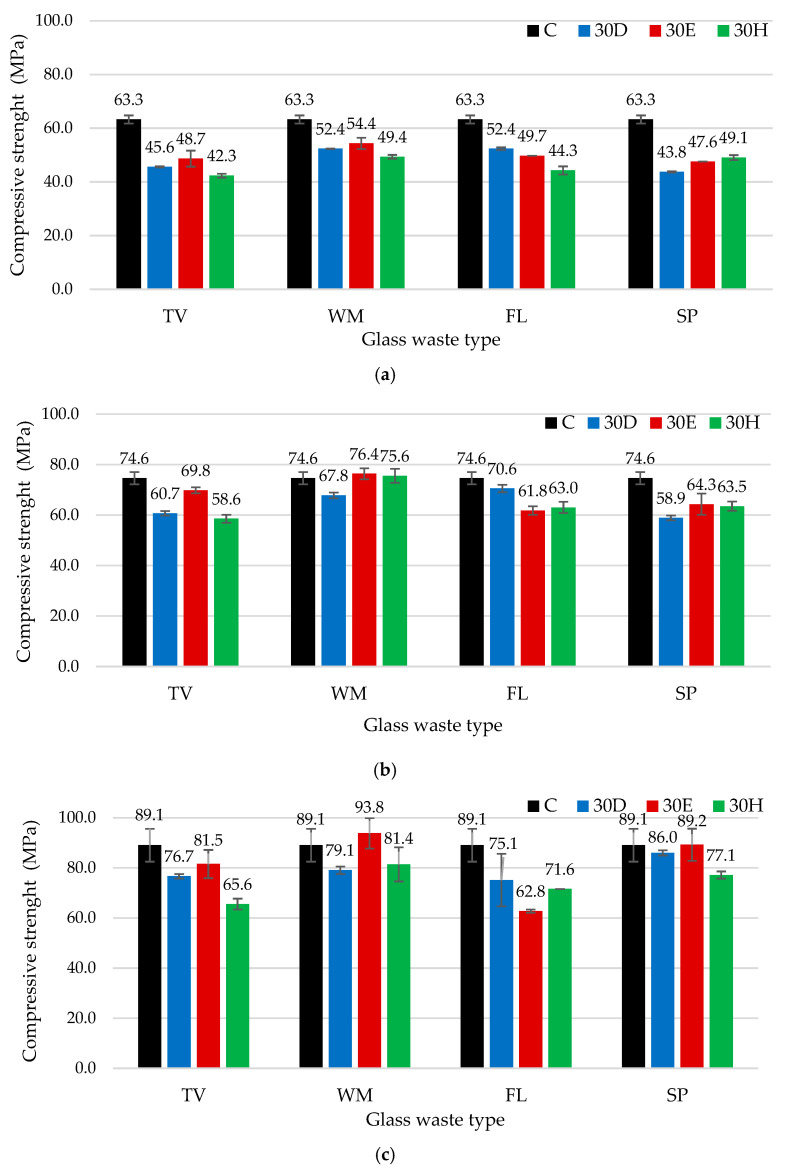
The impact of different mixing methods and GW types on compressive strength: (**a**) after 7 days, (**b**) after 28 days, and (**c**) after 90 days.

Figure 5 and Figure 6 present the results of the binder density and compressive strength after 7, 28 and 90 days. The results of the density test showed that all samples in which cement was partially replaced with GW had lower densities than the control group, regardless of the mixing method or curing time. Depending on the type of glass, the preparation method, and the age of the sample, the decrease in density of the glass-containing samples compared to the control mixture (C) ranged from about 0.5% to almost 6%. The density of the resulting cement samples is primarily influenced by the density of the added glass powders. The TV glass samples prepared by the dispersion method had the highest density among the glass-containing samples in all the periods studied. After 7 days, their density was approximately 0.5% lower than that of the control mixture. After 28 days, the density decrease was approximately 1.6%, and after 90 days, it was only about 0.7%. These results demonstrate that effective dispersion of TV glass particles partially offsets the negative impact of glass on densification during extended hardening periods. The greatest decrease in density was observed in the WM and FL glass samples. After 7 days, the density of the WM glass samples prepared by the dispersion method was about 4.5% lower than that of the control mixture. In the FL glass samples, the density reduction was about 5.9% after 28 days and about 4.9% after 90 days. The lower density of samples with waste glass compared to the control sample may be due to glass particles having a lower density than cement, different particle morphology, possible agglomeration of particles, and slower hydration, all of which influence the compactness and density of the formed microstructure. Overall, these results confirm that, although including glass waste in the binder composition decreases density, the extent of this effect depends greatly on the type of glass and preparation method.

For the TV samples, the increase in density directly correlates with improved paste spread when using either intensive dry mixing or dispersion in water, indicating that the main mechanism in this case is the prevention of particle agglomeration and the enhancement of their uniform distribution. In the case of WM and FL samples, the highest density was obtained using intensive mixing. Although the spread values decreased under these conditions, intensive mixing increases the effective surface area of the particles, thereby promoting enhanced hydration and improved particle packing. Enhanced surface area and reduced interparticle spacing have been shown to facilitate the growth of hydration products and microstructural densification, contributing to higher density in cementitious systems [49]. For SP glass samples, the highest density—although differing by only about 1% (close to the experimental uncertainty)—was obtained using conventional mixing. This result may be attributed to the larger particle size of SP glass and the nearly identical spread results observed across different mixing methods. Larger particles and relatively minor variations in dispersion lead to less sensitivity of the fresh mixture’s rheology to the mixing method and results minimal changes in density [46].

Compressive strength test results (Figure 6) showed that, after 7 days, samples containing GW did not reach the control group values, indicating a negative effect of early hydration regardless of the mixing method used. These results confirm that glass waste limits strength development during the initial hardening period and that the percentage deviation from the control group is negative. As reported in previous scientific studies, the results indicate that, regardless of the type of glass waste used, partial substitution of cement with glass waste during the initials curing period generally retards cement hydration, reduces density, and decreases mechanical strength [50,51]. After 28 days, some samples exceeded the strength of the control group. WM glass samples prepared in an Eirich-type mixer with intensive mixing had a compressive strength about 2.4% higher than the control samples considering that, in this case, 30% of the cement was replaced with a milled waste glass. Meanwhile, WM samples prepared in a Hobart-type mixer exceeded the strength of the control group by about 1.3%. These results demonstrate that, after 28 days, more intensive mixing compensates for the initial loss of strength and achieves values higher than the control samples. After 90 days, the positive effect of intensive mixing became even more pronounced. WM glass samples prepared using intensive mixing achieved a compressive strength that was about 5.3% higher than the control group. However, no such trend was observed in FL and TV glass samples, regardless of the mixing method. Overall, the percentage analysis shows that the intensive mixing method is most favourable for developing compressive strength in GW samples, especially during longer hardening periods. This effect is associated with the more effective dispersion of glass particles, better homogenization of the binder, and more favourable conditions for late hydration and pozzolanic reactions [52]. A comparison of the compressive strength of the same type of glass processed using different mixing methods showed clear differences. After 90 days of curing for TV glass, conventional mixing resulted in the lowest compressive strength (65.6 MPa), whereas intensive mixing yielded the highest value (81.5 MPa), corresponding to a difference of 24.2%. In the case of WM glass, the lowest strength was obtained when the glass was dispersed in water using ultrasound (79.1 MPa), while the highest strength was achieved with intensive mixing (93.8 MPa), resulting in a difference of 18.6%. Only FL glass were the best results obtained when the glass was dispersed in water using ultrasound, which may be attributed to its higher solubility, as indicated by its electrical conductivity being several times higher than that of the other glasses.

**Figure 7:**
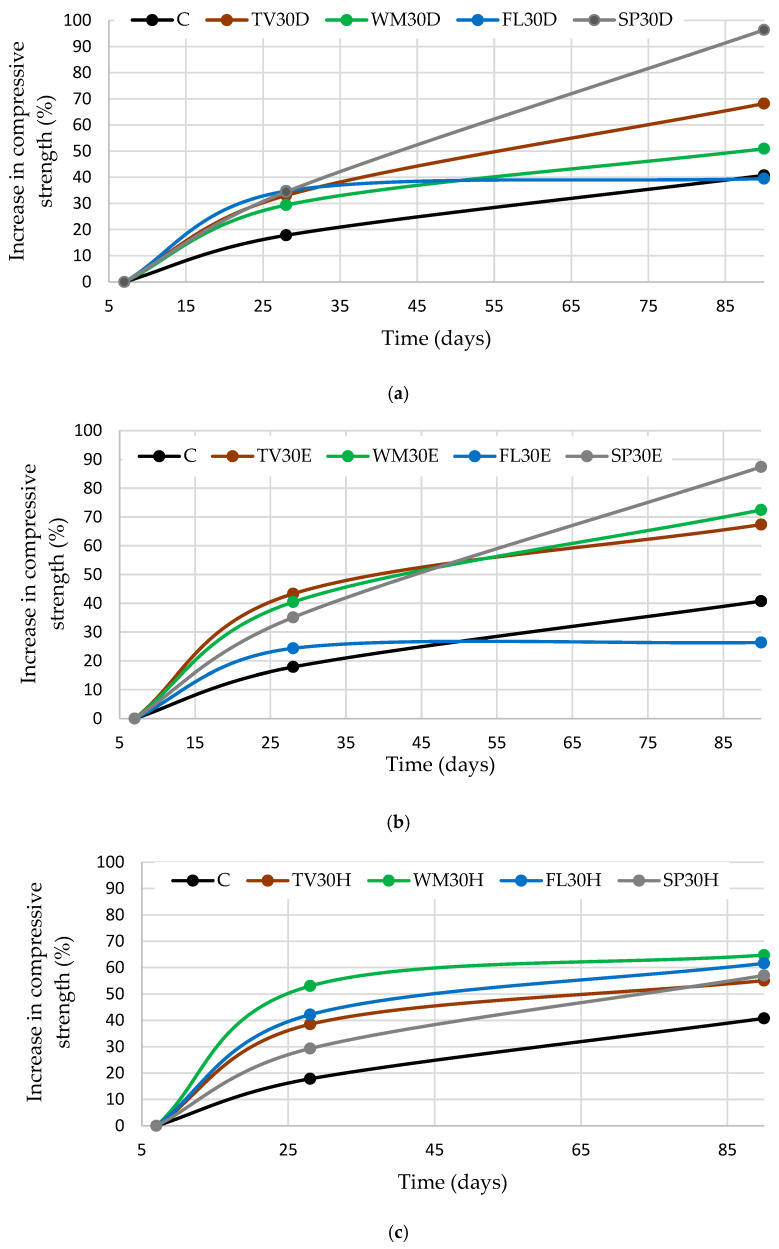
Compressive strength development depending on glass type and mixing method: (**a**) after dispersion with ultrasound, (**b**) after intensive mixing, and (**c**) after conventional mixing.

As compressive strength was found to depend on the type of glass used and the mixing method employed, strength development curves were plotted to evaluate potential strength gains over time (Figure 7). These curves provide a clearer assessment of compressive strength development kinetics between 7 and 90 days, enabling comparison of early-age and long-term performance. The control specimens were found to exhibit the slowest strength development in almost all cases, with an overall increase of approximately 40% between 7 and 90 days. The only exception was the samples prepared using intensive mixing with FL glass, which showed a lower strength gain of 26%. This suggests that, under certain processing conditions, FL glass, which contains the highest amount of sodium oxide, may have a limited contribution to long-term strength development. The enhanced development of early-age strength is attributed to the pozzolanic reactivity of glass, which is primarily associated with its amorphous silica content and the presence of alkali oxides (e.g., Na_2_O). During OPC hydration, a highly alkaline pore solution is generated in which the amorphous glass phase readily dissolves, releasing reactive silica, which reacts with portlandite (Ca(OH)_2_) to form additional calcium silicate hydrate (C-S-H) gel, the main phase responsible for strength in cementitious systems. This mechanism has been widely reported for soda-lime glass powders. The pozzolanic reaction reduces portlandite content and modifies the pore solution chemistry, increasing the concentrations of sodium, silicon and aluminium, and decreasing calcium levels. These changes promote additional C-S-H formation and contribute to the observed enhancement in early compressive strength [53]. However, Na_2_O acts as a network modifier in the glass structure by depolymerising the silicate network and accelerating glass dissolution, thereby altering the hydration pathways. A high alkali content can lead to the rapid early consumption of available calcium, promoting the formation of hydrated phases with a higher sodium content, such as C-(N)-S-H or other sodium-containing amorphous gels. These do not contribute to strength development as effectively as classical C-S-H. While a higher pH accelerates initial glass dissolution and contributes to early pozzolanic activity, it can also limit the availability of free Ca^2+^ ions for continued C-S-H formation at later stages, resulting in a plateau in strength development [54]. Other authors [41] have been reported that glass powder exhibits limited reactivity under the studied conditions and the observed enhancement in cement hydration is therefore not primarily due to the pozzolanic reaction of the glass itself, but rather to the rapid dissolution of alkali ions from the glass surface. These soluble alkalis can increase the pH of the pore solution and accelerate the early stages of cement hydration, leading to a noticeable but indirect effect on the overall reaction process.

The most significant increase in compressive strength was observed in specimens containing SP glass when intensive mixing and dispersion in water were applied. In these cases, compressive strength development followed a roughly linear trend, with an increase of 87–90% when comparing the results at 7 and 90 days. This substantial growth indicates enhanced long-term pozzolanic activity and improved microstructural densification. In contrast, specimens mixed using the conventional method demonstrated exponential strength development. The highest rate of strength gain was recorded within the first 28 days, after which the increase became less pronounced compared to other mixing methods. This behaviour can be explained by faster reaction of easily accessible, reactive glass particles. Meanwhile, agglomerated particles may create localised voids within the matrix, limiting further reaction and slowing down strength development at later ages. When dispersion in water and intensive mixing were applied, glass particles were more uniformly distributed throughout the cementitious matrix. Such distribution likely reduced agglomeration, improved particle–binder contact, and ensured more continuous participation of glass particles in relatively slow pozzolanic reactions. As a result, sustained formation of secondary hydration products contributed to progressive matrix densification and higher long-term compressive strength.

Research has previously established that glass powder has the capacity to diminish the strength of cementitious materials during the initial curing period, which is the first days [35,55,56]. However, at later ages, a marked increase in strength becomes evident, which is attributable to the progression of the pozzolanic reaction. The most substantial improvement in strength is usually observed after 56 or 90 days.

The influence of glass dispersion or mixing methods has been the subject of only a limited number of investigations. As demonstrated in the extant literature [38,39], ground bottle glass was utilised in ultra-high-performance concrete, rather than as a binder component, as examined in the present study. The experimental findings demonstrated that the application of high-frequency ultrasonic dispersion for a duration of 90 s during the process of UHPC mixing resulted in an enhancement of compressive strength by approximately 16%. However, an extension of the duration to 120 s resulted in a decrease of approximately 18%. This phenomenon is presumably associated with the finding that prolonged ultrasonic activation significantly increases the temperature of the fresh mixture and reduces its workability [38]. Our previous studies [37] by analysing microstructure have shown that the reduction in strength is primarily attributed to the agglomeration of fine glass particles. Additional voids form between these particles; over time, these voids become gradually filled with newly formed calcium silicate hydrates, which explains why higher strength is observed after 90 days of curing. However, intensive mixing reduces both the amount and size of agglomerates, leading to a gradual increase in strength, even compared to control specimens, despite a 30% reduction in cement content in the glass-containing mixtures.

### 3.3. Influence of Mixtures Preparation Methods and Waste Glass Types on Binder Mineral Composition and Microstructure

**Figure 8:**
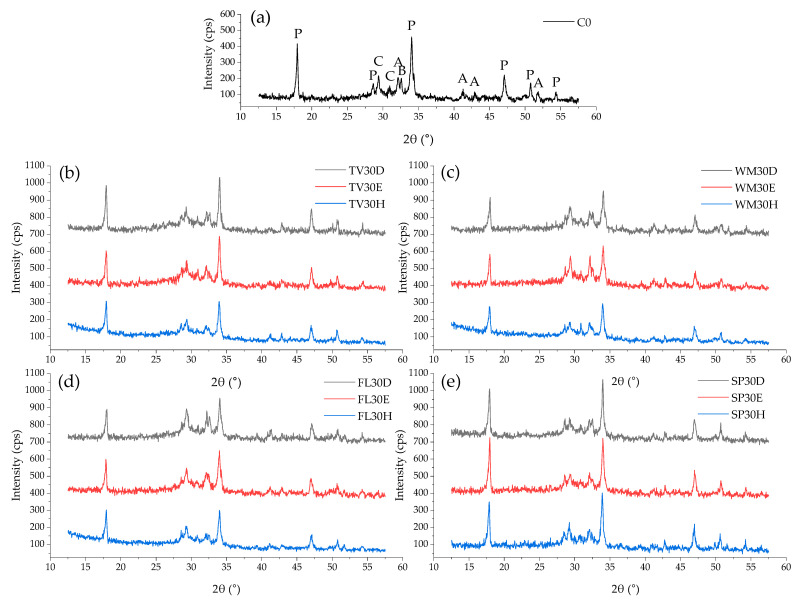
XRD analysis patterns of samples containing different types of GW and obtained by different dispersion methods after 28 days: (**a**)—control sample C0; (**b**) TV glass; (**c**)—WM glass; (**d**)—FL glass; (**e**)—SP glass. P—portlandite, C—calcite, A—alite, B—belite.

**Figure 9:**
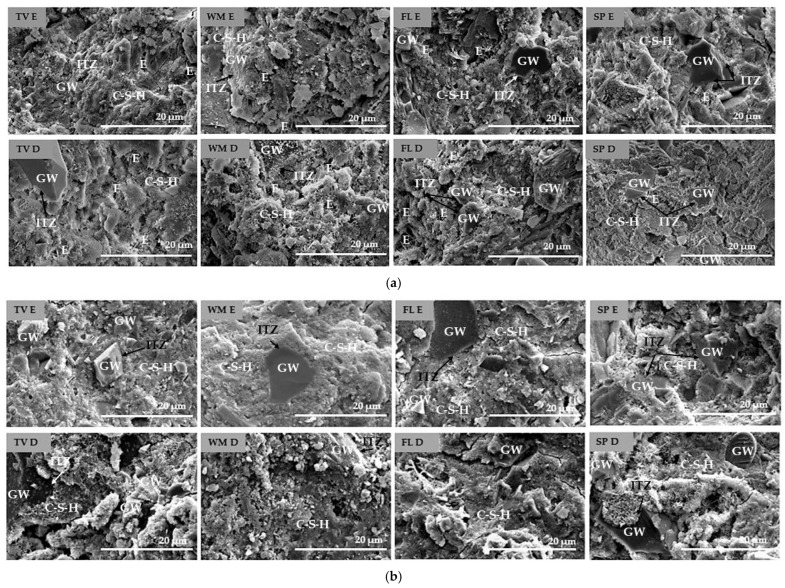
Scanning electron microscopy (SEM) analysis of intensive mixed (E) and dispersed in water with an ultrasound (D), samples after 7 (**a**) and 28 (**b**) days containing different types of glass waste (magnification ×5000): GW—glass waste, C-S-H—calcium silicate hydrate, E—ettringite, ITZ—interaction transition zone.

To identify the causes of the changes in compressive strength, several analytical techniques were used, including XRD (Figure 8) and SEM (Figure 9).

The diffractograms of all mixtures show characteristic peaks of unhydrated clinker minerals (alite and belite), as well as identifiable portlandite and carbonate phases (CaCO_3_). Furthermore, an amorphous hump can also be observed in the 25–32° range, showing existing of C-S-H.

The highest intensity of the cement mineral peaks and newly formed hydration products (especially portlandite) was determined in the control sample due to the highest cement content and the absence of hydration retardation caused by glass powders. In samples containing GW, the intensity of all peaks is lower. Following a 28-day period of observation, XRD analysis demonstrated that the intensity of the primary cement crystalline phases (alite [C_3_S], belite [C_2_S], and the cement hydrate portlandite [Ca(OH)_2_]) is more influenced by the type of GW used than by the method of mixture preparation. However, the peaks of cement hydrates are lowest when glass powder is mixed using the conventional method. In contrast, the intensity of the main diffraction peaks corresponding to the same phases is found to be comparable when intensive mixing is compared with dispersion in water using an ultrasonic method. Nevertheless, some disparities can be identified. As a case in point, in the case of TV glass, the intensity of the portlandite peak is elevated when ultrasonic dispersion is implemented in comparison to intensive mixing. In contrast, for SP glass, the reverse tendency is evident, with augmented portlandite peak intensities being attained subsequent to intensive mixing. WM and FL glass samples demonstrate remarkably analogous portlandite peak intensities following intensive mixing or ultrasonic dispersion in water. A comparison of all GW samples reveals that the lowest peak intensities of portlandite are observed in WM glass samples. Furthermore, these results indicate that lower portlandite peak intensities could be associated with increased formation of C-S-H phases, which in turn leads to higher compressive strength (Figure 6). The highest concentrations of Portlandite were observed in SP samples, in which the coarsest particles were used, and the solubility of such glass is among the lowest. In general, the chemical composition of the glass, its solubility, and its interaction with cement have a significant influence. According to the literature [57], crushed glass (a source of SiO_2_) reacts with portlandite, consuming it and forming additional C-S-H. Thus, the decreasing trend in the intensity of the portlandite peak is a typical indicator of a pozzolanic reaction.

After 28 days of hardening, SEM analysis (Figure 9) revealed that the microstructure of the samples depended significantly on the preparation method and type of glass waste used.

Samples prepared by intensive mixing had a denser binder matrix for all glass types except FL after 7 and after 28 days of curing. This matrix was characterised by a lower number of open pores and a more developed amorphous C-S-H gel. Meanwhile, samples with glass particles dispersed in water (D) usually retained a more porous microstructure in which needle-like and plate-like crystal hydrates, characteristic of ettringite (E) and other early hydration products, were identified. However, an exception was found in FL glass samples, where the prepared samples and the dispersion method formed a relatively denser microstructure compared to D samples of other glass types, because of the highest el. conductivity of this GW—8.21 mS and the highest solubility. Overall, the SEM results confirm that intensive mixing promotes the effective distribution of binder phases and faster C-S-H gel formation. In contrast, the dispersion method often results in the predominance of locally crystallised hydration products and higher porosity [58].

## 4. Conclusions

- The method of mixing raw materials was determined to have a significant effect on the rheological properties of cement-based binders with ground waste glass, depending on the type of glass. The ultrasonic dispersion technique was found to be most effective for low-soluble TV glass, yielding spread values that were comparable to those of the reference mixture due to enhanced particle dispersion. The findings of the study suggest that while WM and FL glass powders exhibit comparable particle sizes to those of TV, their responses to dispersion methods differ. The application of intensive mixing did not result in an enhancement of paste spread; indeed, it led to a reduction in spread, particularly in the case of WM. Conversely, ultrasonic dispersion yielded only a marginal increase in spread. This behaviour is attributed to the higher solubility of these glasses. It has been established that dissolution processes at the glass–cement interface are conducive to the formation of a hydrated amorphous gel layer. Consequently, glass solubility plays a pivotal role in the control of dispersion efficiency and paste rheology.
- The findings indicate that the density and compressive strength of cement-based binders are contingent on both the type of glass and the method of mixing employed. The highest density for TV glass was achieved by dispersion in water, while intensive mixing was most effective for WM and FL glass, likely due to enhanced hydration and microstructural densification. The effect was negligible for SP glass. Intensive mixing was found to be optimal for TV and WM glass, while ultrasonic dispersion yielded the best results for FL glass due to the highest solubility, highlighting the need to tailor mixing methods to glass properties. The results of XRD and SEM analyses demonstrated that the mixing methods employed had a significant impact on both hydration and microstructure. Intensive mixing generally produced a denser matrix, whereas ultrasonic dispersion resulted in a more porous structure, with the exception of FL glass, which exhibited the highest solubility.

## Figures and Tables

**Table 1:** The chemical composition of cement and different glass waste.

Chemical Component (%)	Cement	TV	WM	FL	SP
SiO_2_	20.0	53.1	81.4	67.9	69.4
Al_2_O_3_	4.57	2.30	2.91	2.11	1.12
Na_2_O	0.14	6.37	6.51	14.2	12.2
K_2_O	1.30	7.85	1.26	1.75	0.04
MgO	3.71	0.31	0.03	2.54	3.24
CaO	63.4	0.93	3.74	5.45	9.96
BaO	0.06	8.62	0.03	1.38	0.04
PbO	-	3.98	-	0.10	0.01
SrO	-	7.70	0.01	0.06	0.01
ZnO	0.01	0.36	-	-	0.002
Other	6.81	8.48	4.11	4.51	3.98

## Data Availability

The original contributions presented in this study are included in the article. Further inquiries can be directed to the corresponding author.

## Abbreviations

The following abbreviations are used in this manuscript:

GW	Glass waste
TV	Discarded television screens
WM	Washing machines
FL	Fluorescent lamps
SP	Photovoltaic solar panels
C-S-H	Calcium silicate hydrates
